# Infarct size, inflammatory burden, and admission hyperglycemia in diabetic patients with acute myocardial infarction treated with SGLT2-inhibitors: a multicenter international registry

**DOI:** 10.1186/s12933-022-01506-8

**Published:** 2022-05-15

**Authors:** Pasquale Paolisso, Luca Bergamaschi, Gaetano Santulli, Emanuele Gallinoro, Arturo Cesaro, Felice Gragnano, Celestino Sardu, Niya Mileva, Alberto Foà, Matteo Armillotta, Angelo Sansonetti, Sara Amicone, Andrea Impellizzeri, Gianni Casella, Ciro Mauro, Dobrin Vassilev, Raffaele Marfella, Paolo Calabrò, Emanuele Barbato, Carmine Pizzi

**Affiliations:** 1 Cardiovascular Center Aalst, OLV-Clinic, Aalst, Belgium; 2 Department of Advanced Biomedical Sciences, University Federico II, Naples, Italy; 3 Unit of Cardiology, Department of Experimental, Diagnostic and Specialty Medicine-DIMES, University of Bologna, 40138 Bologna, Italy; 4 International Translational Research and Medical Education (ITME) Consortium, Naples, Italy; 5 Department of Medicine (Division of Cardiology) and Department of Molecular Pharmacology, Wilf Family Cardiovascular Research Institute, Einstein-Sinai Diabetes Research Center, The Fleischer Institute for Diabetes and Metabolism, Albert Einstein College of Medicine, New York, USA; 6 Department of Translational Medical Sciences, University of Campania ‘Luigi Vanvitelli’, Naples, Italy; 7 Department of Translational Medical Sciences, University of Campania “Luigi Vanvitelli”, Naples, Italy; 8 Division of Cardiology, A.O.R.N. “Sant’Anna e San Sebastiano”, Caserta, Italy; 9 Department of Advanced Medical and Surgical Sciences, University of Campania “Luigi Vanvitelli”, Naples, Italy; 10 Cardiology Clinic, ″Alexandrovska″ University Hospital, Medical University of Sofia, Sofia, Bulgaria; 11 Unit of Cardiology, Maggiore Hospital, Bologna, Italy; 12 Department of Cardiology, Hospital Cardarelli, Naples, Italy; 13 Medica Cor Hospital, Russe, Bulgaria; 14 Mediterranea Cardiocentro, Naples, Italy

**Keywords:** SGLT2-I, Hyperglycemia, Inflammation, Infarct size, Acute myocardial infarction

## Abstract

**Background:**

The inflammatory response occurring in acute myocardial infarction (AMI) has been proposed as a potential pharmacological target. Sodium-glucose co-transporter 2 inhibitors (SGLT2-I) currently receive intense clinical interest in patients with and without diabetes mellitus (DM) for their pleiotropic beneficial effects. We tested the hypothesis that SGLT2-I have anti-inflammatory effects along with glucose-lowering properties. Therefore, we investigated the link between stress hyperglycemia, inflammatory burden, and infarct size in a cohort of type 2 diabetic patients presenting with AMI treated with SGLT2-I versus other oral anti-diabetic (OAD) agents.

**Methods:**

In this multicenter international observational registry, consecutive diabetic AMI patients undergoing percutaneous coronary intervention (PCI) between 2018 and 2021 were enrolled. Based on the presence of anti-diabetic therapy at the admission, patients were divided into those receiving SGLT2-I (SGLT-I users) versus other OAD agents (non-SGLT2-I users). The following inflammatory markers were evaluated at different time points: white-blood-cell count, neutrophil-to-lymphocyte ratio (NLR), platelet-to-lymphocyte ratio (PLR), neutrophil-to-platelet ratio (NPR), and C-reactive protein. Infarct size was assessed by echocardiography and by peak troponin levels.

**Results:**

The study population consisted of 583 AMI patients (with or without ST-segment elevation): 98 SGLT2-I users and 485 non-SGLT-I users. Hyperglycemia at admission was less prevalent in the SGLT2-I group. Smaller infarct size was observed in patients treated with SGLT2-I compared to non-SGLT2-I group. On admission and at 24 h, inflammatory indices were significantly higher in non-SGLT2-I users compared to SGLT2-I patients, with a significant increase in neutrophil levels at 24 h. At multivariable analysis, the use of SGLT2-I was a significant predictor of reduced inflammatory response (OR 0.457, 95% CI 0.275–0.758, p = 0.002), independently of age, admission creatinine values, and admission glycemia. Conversely, peak troponin values and NSTEMI occurrence were independent predictors of a higher inflammatory status.

**Conclusions:**

Type 2 diabetic AMI patients receiving SGLT2-I exhibited significantly reduced inflammatory response and smaller infarct size compared to those receiving other OAD agents, independently of glucose-metabolic control. Our findings are hypothesis generating and provide new insights on the cardioprotective effects of SGLT2-I in the setting of coronary artery disease.

*Trial Registration:* Data are part of the ongoing observational registry: SGLT2-I AMI PROTECT. ClinicalTrials.gov Identifier: NCT 05261867.

## Background

Among patients with acute myocardial infarction (AMI), various pathophysiological events occur due to ischemia and generate an intense inflammatory response [1, 2]. Furthermore, restoring blood flow produces a ‘second hit’ phenomenon, called ischemia-reperfusion (I/R) injury, more remarkable than the primary ischemic event. The I/R injury results from combined events, including production of reactive oxygen species (ROS) and inflammation [3]. Neutrophils are the first leukocytes detected in infarcted areas, followed by monocytes and lymphocytes, releasing proteo-enzymes and cytokines, and phagocytizing necrotic debris [4, 5]. Mounting evidence suggests that neutrophil-to-lymphocyte ratio (NLR), platelet-to-lymphocyte ratio (PLR), and neutrophil-to-platelet ratio (NPR) might be considered as biomarkers of systemic inflammation and associated with poor clinical outcomes in various cardiovascular diseases, including acute coronary syndromes (ACS) [1, 6–8]. Moreover, recent investigations have shown that the inflammatory status correlates with infarct size and adverse clinical outcome in ACS patients [9, 10].

Sodium-glucose cotransporter 2 inhibitors (SGLT2-I) are oral antidiabetic (OAD) agents that exert beneficial effects on glycemic control in type 2 diabetes mellitus (T2DM). In large, randomized trials, SGLT2-I significantly improved cardiovascular and renal outcomes in diabetic patients, with benefits extended to non-diabetic patients with and without heart failure [11–13]. In addition, SGLT-2-I have been tested in several preclinical studies demonstrating the reduction in acute myocardial I/R injury [14]. Based on these observations, we hypothesized that SGLT2-I might have cardio-protective and anti-inflammatory effects independently of their anti-hyperglycemic properties [15, 16]. To test this hypothesis, we investigated the inflammatory burden and myocardial infarct size in T2DM patients with AMI receiving SGLT2-I compared to other OAD agents (non-SGLT-I users).

## Methods

### Study population

In this multicenter international observational registry (SGLT2-I AMI PROTECT, ClinicalTrials.gov Identifier: NCT 05261867), we screened consecutive diabetic patients admitted with AMI, both ST-segment elevation myocardial infarction (STEMI) and non-ST-segment elevation myocardial infarction (NSTEMI), undergoing percutaneous coronary intervention (PCI), between January 2018 and September 2021. The definition of STEMI and NSTEMI and patients’ management followed current guidelines [17, 18]. Based on admission antidiabetic therapy, patients were divided into SGLT2-I users, if they were admitted on chronic SGLT2-I therapy (i.e., started at least 3 months before hospitalization), and non-SGLT2-I users, if they received other OAD strategies alone. Patients on insulin therapy or with incomplete information on medical therapy were excluded. Further exclusion criteria were AMI treated with coronary artery bypass grafting, severe valvular heart disease, prosthetic heart valves, severe anemia, history of or ongoing bleeding, pulmonary embolism, fever (≥ 38 °C), chronic renal failure (glomerular filtration rate < 30 mL/min/1.73 m^2^), autoimmune diseases, malignancies or ongoing cardiotoxic medications, and congenital heart disease. Patients with more than 20% of missing values in the collected data were also excluded due to potential bias. The present study was conducted according to the principles of the Declaration of Helsinki; all patients were informed about their participation in the registry and provided informed consent for the anonymous publication of scientific data.

### Inflammatory biomarkers and infarct size detection

Systemic inflammatory markers [C-reactive protein (CRP), white-blood-cell count (WBC) and neutrophils count] were determined according to standard protocols, on admission and after 24 h. The inflammatory response was evaluated using the following parameters: total white blood cells, NLR, NPR, PLR and CRP. Patients with concomitant basal values of CRP and NLR above the median of the study population were considered to have an inflammatory response. For all patients, blood for high-sensitivity Troponin I (hs-TnI) evaluation was drawn at the time of hospital admission and every 3–6 h thereafter for the following 24 h. The hs-TnI peak was considered the highest value before its fall.

All patients underwent a 2D echocardiogram at admission and before discharge, performed by experienced operators. At least 3 consecutive beats were recorded for each view, and all images were stored for offline analysis. Left ventricular ejection fraction (LVEF) was calculated with the biplane Simpson’s method according to the European Association of Cardiovascular Imaging guidelines [19]. Myocardial infarct size was estimated using the left ventricular end-diastolic volume (LVEDV), the biplane LVEF and the regional wall motion abnormalities (RWMA) defined as having at least two hypokinetic or akinetic segments with or without LVEF < 50%. Wall motion abnormalities were visually assessed based on the observed wall thickening and endocardial motion of the myocardial segment according to the American Society of Echocardiography and the European Association of Cardiovascular Imaging guidelines [19].

### Blood glucose and definition of diabetes mellitus

Blood glucose levels were assessed on admission as part of the standard evaluation. Pre-existing T2DM was defined as known DM at the time of hospitalization irrespective of the therapeutic management (diet and lifestyle measures alone or additional administration of oral glucose-lowering medication and insulin) [20].

### Statistical analysis

Data distribution was assessed visually with histograms or the Shapiro-Wilk test as appropriate. Differences between groups were analyzed using the t-test or the Mann–Whitney U-test for continuous variables and the chi-square test or the Fisher’s exact test for categorical variables, as appropriate. Continuous variables were summarized using the mean and standard deviation or median and interquartile range, as appropriate. A multiple logistic regression model was used to identify independent predictors of inflammation. Correlation between variables was assessed with either Pearson’s R or Spearman’s ρ, as appropriate. In addition, linear and polynomial regression models were fit to evaluate the relationship between continuous variables. All analyses were performed using the Statistical Package for Social Sciences, version 25.0 (SPSS, PC version, IBM Corp, Armonk, NY, USA) and R version 3.5.2 (R Foundation for Statistical Computing, Vienna, Austria). The significance level was set to p < 0.05.

## Results

### Study population

Out of 993 AMI diabetic patients screened, 286 were excluded due to insulin therapy, 113 because of coronary artery bypass grafting and 11 for all the others exclusion criteria. The final study population consisted of 583 diabetic AMI patients treated with PCI, which were divided in SGLT2-I (n = 98) or non-SGLT2-I users (n = 485).

### Baseline characteristics

Baseline characteristics and medical therapy on admission are reported in Table 1. The mean age of the overall study population was 71 years, and more than 76.2% were males. SGLT2-I patients were younger and presented better renal function on admission compared to non-SGLT2-I users. The mean time of exposure to SGLT2-I therapy was 7.3 $\pm$ 3.1 months. At variance, gender, body mass index/surface area, main cardiovascular risk factors, glucose-metabolic control, and comorbidities were similar in the two groups. Regarding medical therapy at the admission, no differences were found, except for a lower intake of sulfonylureas in SGLT2-I users **(**Table 1**)**.

**Table 1:** Comorbidities and admission medical therapy

	Total (N = 583)	SGLT2-I users (N = 98)	Non-SGLT-I users (N = 485)	p value
Age, n (%)	71 [61–79]	65 [58–74]	72 [62–80]	< 0.001
Male Sex, n (%)	444 (76.2)	80 (81.6)	364 (75.1)	0.163
BMI, Kg/m2	28.3 ± 4.9	28.2 ± 4.9	28.4 ± 5	0.577
BSA, m2	1.9 ± 0.2	1.9 ± 0.3	1.9 ± 0.2	0.145
Smoking, n (%)	337 (57.8)	62 (63.3)	275 (56.7)	0.230
Hypertension, n (%)	485 (83.2)	87 (88.8)	398 (82.1)	0.105
Dyslipidemia, n (%)	460 (78.9)	84 (85.7)	376 (77.5)	0.07
PAD, n (%)	70 (12)	13 (13.3)	57 (11.8)	0.674
COPD, n (%)	78 (13.4)	13 (13.3)	65 (13.4)	0.971
CKD, n (%)	51 (8.7)	8 (8.2)	43 (8.9)	0.822
Previous TIA/CVA, n (%)	46 (7.9)	9 (9.2)	37 (7.6)	0.603
Previous AMI, n (%)	152 (26.1)	29 (29.6)	123 (25.4)	0.384
Previous PCI, n (%)	166 (28.5)	33 (33.7)	133 (27.4)	0.211
AF, n (%)	53 (9.1)	9 (9.2)	44 (9.1)	0.972
Antiplatelets, n (%)	293 (50.3)	52 (53.1)	241 (49.7)	0.543
Anticoagulation, n (%)	50 (8.6)	5 (5.1)	45 (9.3)	0.178
RAAS-I, n (%)	330 (56.6)	64 (65.3)	266 (54.8)	0.07
Diuretics, n (%)	170 (32)	26 (26.5)	144 (29.7)	0.820
B-blockers, n (%)	255 (43.7)	47 (48)	208 (42.9)	0.356
CCB, n (%)	156 (26.8)	22 (22.4)	134 (27.6)	0.291
Statins, n (%)	284 (48.7)	54 (55.1)	230 (47.4)	0.165
Low/moderate intensity	203 (71.5)	35 (64.8)	173 (75.2)	0.120
High intensity	81 (28.5)	19 (35.2)	57 (24.8)	
Ezetimibe, n (%)	70 (12)	13 (13.3)	57 (11.8)	0.674
Metformin, n (%)	420 (72)	72 (73.5)	348 (71.8)	0.730
Sulfonylureas, n (%)	157 (26.9)	12 (12.2)	145 (30)	0.001
DPP-4 Inhibitors, n (%)	47 (8.1)	7 (7.1)	40 (8.2)	0.714
GLP-1 Agonist, n (%)	14 (2.4)	3 (3.1)	11 (2.3)	0.640

Continuous variables are presented as mean ± SD or as median [IQR]; categorical variables as number (%). BMI:  Body Mass Index; BSA:  Body Surface Area; PAD: peripheral arterial disease; COPD: Chronic obstructive pulmonary disease; CKD: Chronic kidney disease with 30 < GFR < 60 ml/min; PCI:  Percutaneous Coronary Intervention; AF: Atrial fibrillation; RAAS-I: Renin-angiotensin-aldosterone system inhibitors; CCB: Calcium Channel Blockers; DPP-4: Dipeptidyl Peptidase 4; GLP-1: Glucagon-like peptide-1

The two study groups exhibited similar admission characteristics, including GRACE risk score, and Killip class, except for admission heart rate that was significantly lower in SGLT2-I users than in non-SGLT2-I patients (Table 2**)**. Rate of STEMI was similar between the two subgroups. The median times from symptoms to diagnostic coronary angiography did not differ between groups for both STEMI and NSTEMI **(**Table 2**)**. Finally, the main angiographic characteristics were also similar between the two study groups (Table 2).

**Table 2:** Clinical admission and angiographic characteristics

	Total (N = 583)	SGLT2-I users (N = 98)	Non-SGLT-I users (N = 485)	P value
STEMI, n (%)	279 (47.9)	48 (49)	231 (47.6)	0.807
Time symptoms–balloon (STEMI)	3[2–5]	3[2–6]	3 [2– 5]	0.756
Time symptoms–balloon < 24 h (NSTEMI)	188 (61.8)	34 (68)	154 (60.6)	0.326
SBP, mmHg	140 [125–160]	140 [125–151]	140 [125–160]	0.501
DBP, mmHg	80 [70–90]	84 [70–90]	80 [70–90]	0.364
HR	81 [70–94]	75 [65–86]	83 [72–95]	< 0.001
Angina, n (%)	427 (73.2)	70 (71.4)	357 (73.6)	0.657
Killip Class ≥ 2, n (%)	119 (20.4)	14 (14.3)	105 (21.6)	0.099
GRACE Score	156 ± 38	152 ± 36	156 ± 38	0.603
LM lesion, n (%)	28 (4.8)	2 (2)	26 (5.4)	0.161
LAD lesion, n (%)	332 (56.9)	58 (59.2)	274 (56.5)	0.624
CX lesion, n (%)	150 (25.7)	28 (28.6)	122 (25.2)	0.480
RCA lesion, n (%)	182 (31.2)	30 (30.6)	152 (31.3)	0.887
1 Vessel lesion, n (%)	250 (42.9)	48 (49)	202 (41.6)	0.181
2 Vessels lesion, n (%)	205 (35.2)	31 (31.6)	174 (35.9)	0.422
3 Vessels lesion, n (%)	124 (21.3)	17 (17.3)	107 (22.1)	0.298
Hospital stays, days	5 [4–8]	5 [4–7]	5 [4–8]	0.896

Continuous variables are presented as median (IQR) while categorical ones as n (%). STEMI: ST-segment Elevation Myocardial Infarction; NSTEMI:  non-ST segment Elevation Myocardial Infarction; SBP: Systolic blood pressure; DBP:  Diastolic blood pressure; HR: Heart rate; LM : Left main; LAD: Left anterior descending artery; CX: Circumflex artery; RCA: Right coronary artery

### Impact of SGLT2-I on infarct size

Infarct size parameters are shown in Table 3. On admission, left ventricular volume and function and RMWA were similar between the two study groups. The individual values of the troponin curve, as well as the peak troponin values, ​​were significantly lower in SGLT2-I users than non-SGLT2-I patients (p ≤ 0.003 for all, Table 3). Consistently, ST-segment resolution post-PCI was more frequent in SGLT2-I group (p = 0.001). Likewise, the infarct size measured by left ventricular function and RMWA at discharge was significantly lower in the SGLT2-I group compared to non-SGLT2-I users (p = 0.001 for both).

**Table 3:** Infarct size in patients with SGLT2-I versus patients with other OAD agents alone

	Total (N = 583)	SGLT2-I users (N = 98)	Non-SGLT-I users (N = 485)	p value
Hospital Admission				
Q wave, n (%)	131 (25.2)	18 (23.4)	113 (25.6)	0.615
Admission LVEDV, ml	108 ± 33	106 ± 35	108 ± 33	0.582
Admission LVEF, %	47 ± 11	48 ± 10	47 ± 11	0.161
RWMA, n (%)	491 (84.2)	81 (82.7)	410 (84.5)	0.641
I hs-TnI, ng/L	210 [44–1431]	131 [33–773]	240 [50–1964]	0.003
II hs-TnI, ng/L	1411 [338–10.032]	635 [165–2108]	1842 [370–13.447]	< 0.001
III hs-TnI, ng/L	1306 [390–11.028]	441 [160–1120]	2356 [566–18.056]	< 0.001
hs-TnI max, ng/L	2438 [591–16.227]	901 [307–2543]	3445 [710–9223]	< 0.001
Hospital Discharge				
LVEDV, ml	108 ± 36	103 ± 29	110 ± 38	0.261
LVEF, %	49 ± 10	53 ± 19	48 ± 11	0.001
RWMA, n (%)	454 (78)	64 (65.3)	390 (80.6)	0.001
ST resolution, n (%)	187 (67)	42 (87.5)	146 (63.2)	0.001

Continuous variables are presented as median (IQR) while categorical ones as n (%). Hs-TnI: High sensitivity Troponin; LVEDV: Left ventricular end diastolic volume; LVEF: Left ventricular ejection fraction; RWMA: Regional wall motion abnormalities

### Impact of SGLT2-I on the inflammatory burden

**Fig. 1:**
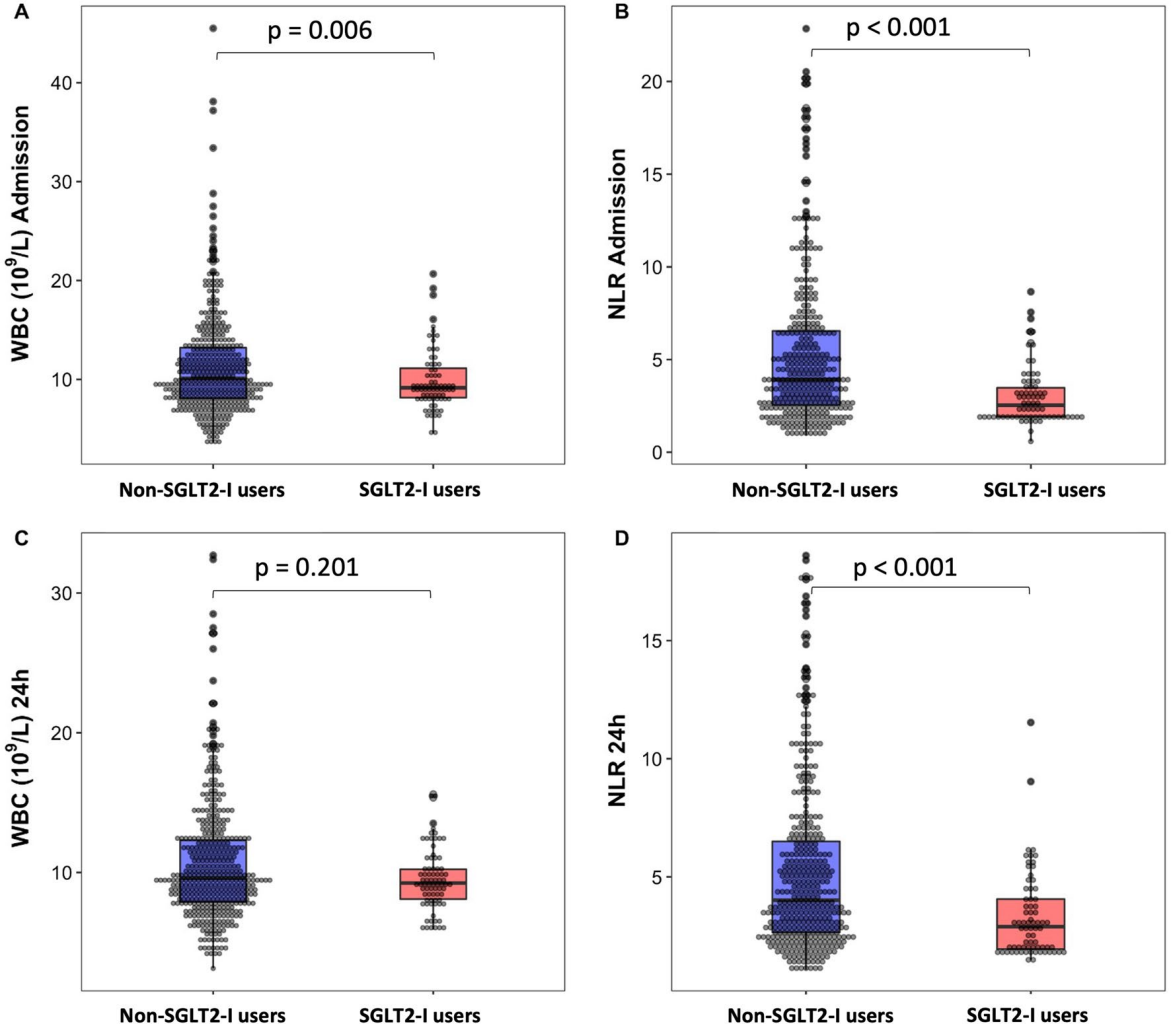
Box plot and Dot plot comparing the distribution of White Blood Cells (**A** and **C**) and Neutrophil-Lymphocyte Ratio (NLR) (**B** and **D**) at admission and discharge in SGLT2-I users vs. non-SGLT2-I users (blue box and red box respectively)

**Fig. 2:**
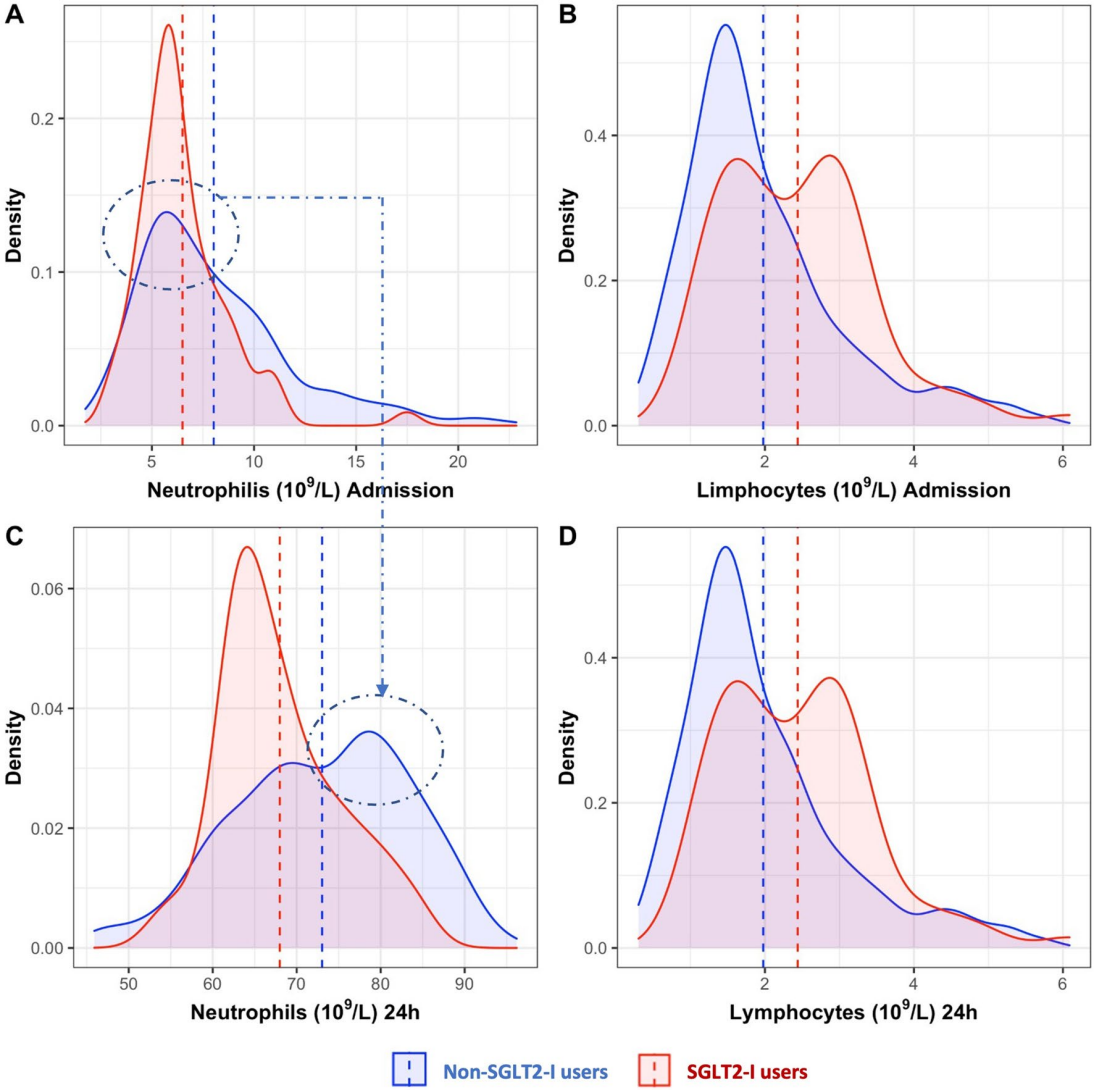
Density Plot showing the neutrophils (**A** and **C**) and lymphocytes (**B** and **D**) distribution at admission and after 24 h in SGLT2-I versus non-SGLT2-I users. Blue curve denotes non-SGLT2-I users; red curve represents patients receiving SGLT2-I. The dotted arrow shows how the peak of the neutrophil’s distribution in non SGLT2-I users moved towards higher values after 24 h

Inflammatory markers are presented in Table 4. On admission, total WBC count, neutrophils, and CRP levels were significantly higher in non-SGLT2-I patients compared to those receiving SGLT2-I (p < 0.006 for all). Consequently, the NLR, PLR, and NPR were markedly lower in the SGLT2-I users (p < 0.03 for all, Fig. 1). After 24 h, neutrophils, NLR and CRP remained significantly lower in patients receiving SGLT2-I (p = 0.03, p < 0.001 and p = 0.04, respectively). Furthermore, CRP values remained higher in non-SGLT2-I users at discharge (p = 0.01). As shown in Fig. 2, trends in lymphocyte and neutrophil levels were markedly different between the two groups: a significant increase in neutrophil levels at 24 h were observed in non-SGLT2-I patients but not in the SGLT2-I group. The admission blood glucose levels, but not glycosylated hemoglobin (HbA1c), were significantly lower in SGLT2-I-patients compared to non-SGLT2-I group (p = 0.006).

**Table 4:** Laboratory data and biomarkers in patients with SGLT2-I versus patients with other OAD agents alone

	Total (N = 583)	SGLT2-I users (N = 98)	Non-SGLT-I users (N = 485)	p value
Hospital Admission				
WBC, 10^9^/L	9.7 [8.1–12.8]	9 [8.1–10.8]	10 [8.1–13.1]	0.006
Neutrophils, 10^9^/L	6.8 [5.3–9.4]	6 [5.3–7.5]	7.1 [5.3–9.8]	0.001
Lymphocytes, 10^9^/L	1.8 [1.3–2.6]	2.2 [1.5–2.9]	1.7 [1.3–2.5]	0.001
PLTs, count x 10^9^ per L	241 [187–290]	230 [178–292]	243 [189–291]	0.207
NLR	3.6 [2.4–5.9]	2.9 [1.9–4.1]	3.9 [2.5–6.5]	< 0.001
PLR	126.2 [91.5–175.8]	102.5 [77.7–143.2]	132.1 [95–182.3]	< 0.001
NPR	0.03 ± 0.02	0.03 ± 0.01	0.03 ± 0.02	0.03
CPR, mg/dL	3.9 ± 12.6	3.1 ± 3.8	4 ± 13.8	< 0.001
Adm. Creatinine, mg/dL	1.2 ± 0.9	1.03 ± 0.3	1.2 ± 0.9	0.016
HbA1c, mmol/L	50 [46–58]	52 [48–56]	50 [44–59]	0.583
Admission Glycemia, mg/dl	180 [144–240]	156 [139–200]	187 [147–247]	0.006
BNP, pg/ml	378 [118–1015]	235 [85–452]	474 [129–1306]	< 0.001
After 24 h				
WBC, N/µl	9.5 [7.9–12]	9.2 [8.1–10.4]	9.5 [7.9–12.3]	0.201
Neutrophils, 10^9^/L	6.7 [5.4–9.1]	6.2 [5.5–7.3]	7 [5.3–9.6]	0.03
Lymphocytes, 10^9^/L	1.7 [1.2–2.4]	2 [1.4–3.1]	1.7 [1.2–2.3]	0.001
NLR	3.8 [2.5–6]	3 [1.9–4.6]	4 [2.7–6.6]	< 0.001
CPR, mg/dL	9 ± 21	3.3 ± 4.4	11 ± 23.5	0.040
Hospital Discharge				
WBC, N/µl	8.5 [7–9.8]	8.6 [7.4–9.5]	8.4 [6.9–9.8]	0.587
CPR, mg/dL	8 ± 24.3	3.1 ± 3.7	9.9 ± 28.5	0.01

Continuous variables are presented as median (IQR) while categorical ones as n (%). WBC:   White blood cell; PLTs: Platelets; NLR: Neutrophil-to-lymphocyte ratio; PLR: Platelet-to-lymphocyte ratio; NPR: Neutrophil-to-platelet ratio; CRP:  C-reactive protein

At multivariable analysis, the use of SGLT2-I was a significant predictor of reduced inflammatory response (OR 0.457, 95% CI 0.275–0.758, p = 0.002), independently of age, admission creatinine values, and admission glycemia (Table 5). Conversely, peak troponin values (OR 1.000, 95% CI 1.001–1.002, p = 0.025) and NSTEMI occurrence (OR 1.702, 95% CI 1.129–2.566, p = 0.011) were independent predictors of a higher inflammatory status.

**Table 5:** Multivariable analysis – Predictors of inflammatory response

Variables	Std. Err.	OR	95% CI	p-value
Age, years	0.009	1.011	0.994–1.028	0.197
Adm. Creatinine, mg/dL	0.152	1.333	0.990–1.796	0.060
Admission glycemia, mg/dL	0.001	1.002	1.000–1.004	0.100
NSTEMI	0.209	1.702	1.129–2.566	0.011
hs-TnI max, ng/L	0.001	1.008	1.001–1.015	0.025
SGLT2-I	0.259	0.457	0.275–0.758	0.002

**Fig. 3:**
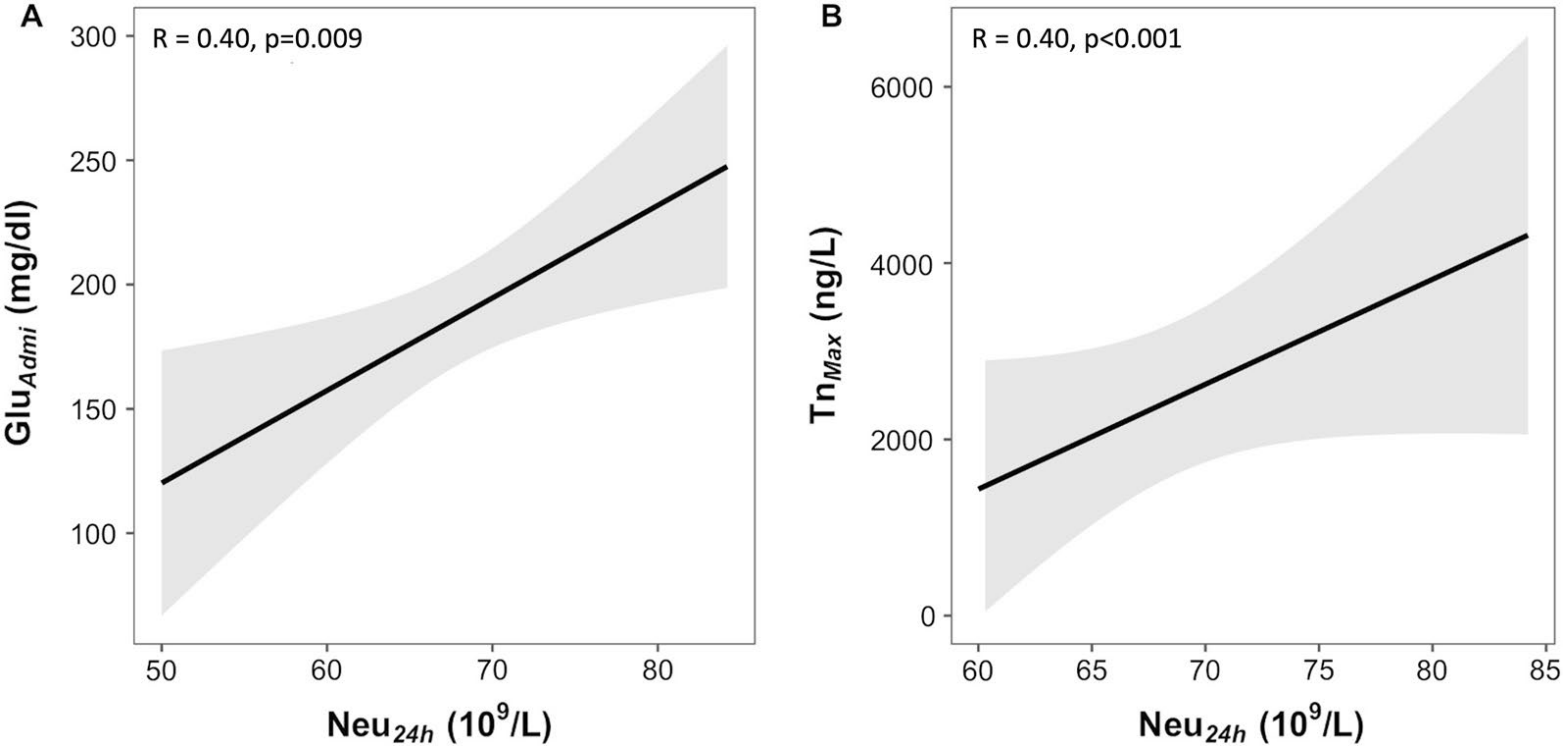
Correlations between neutrophils values measured at 24 h and the admission glucose levels (**A**) and peak troponin values (**B**), in the overall study population (n = 583)

In the overall study population (n = 583), positive linear correlations were found between neutrophils values measured at 24 h and both the admission glucose levels (r = 0.40, p = 0.009) and peak troponin values (r = 0.40, p < 0.001) (Fig. 3**)**. Notably, neutrophil values and peak troponin values were confirmed to be linearly correlated independently of the admission glucose level (r = 0.31, p < 0.001), supporting the effect of other mechanisms in addition to glucose-metabolic control.

**Fig. 4:**
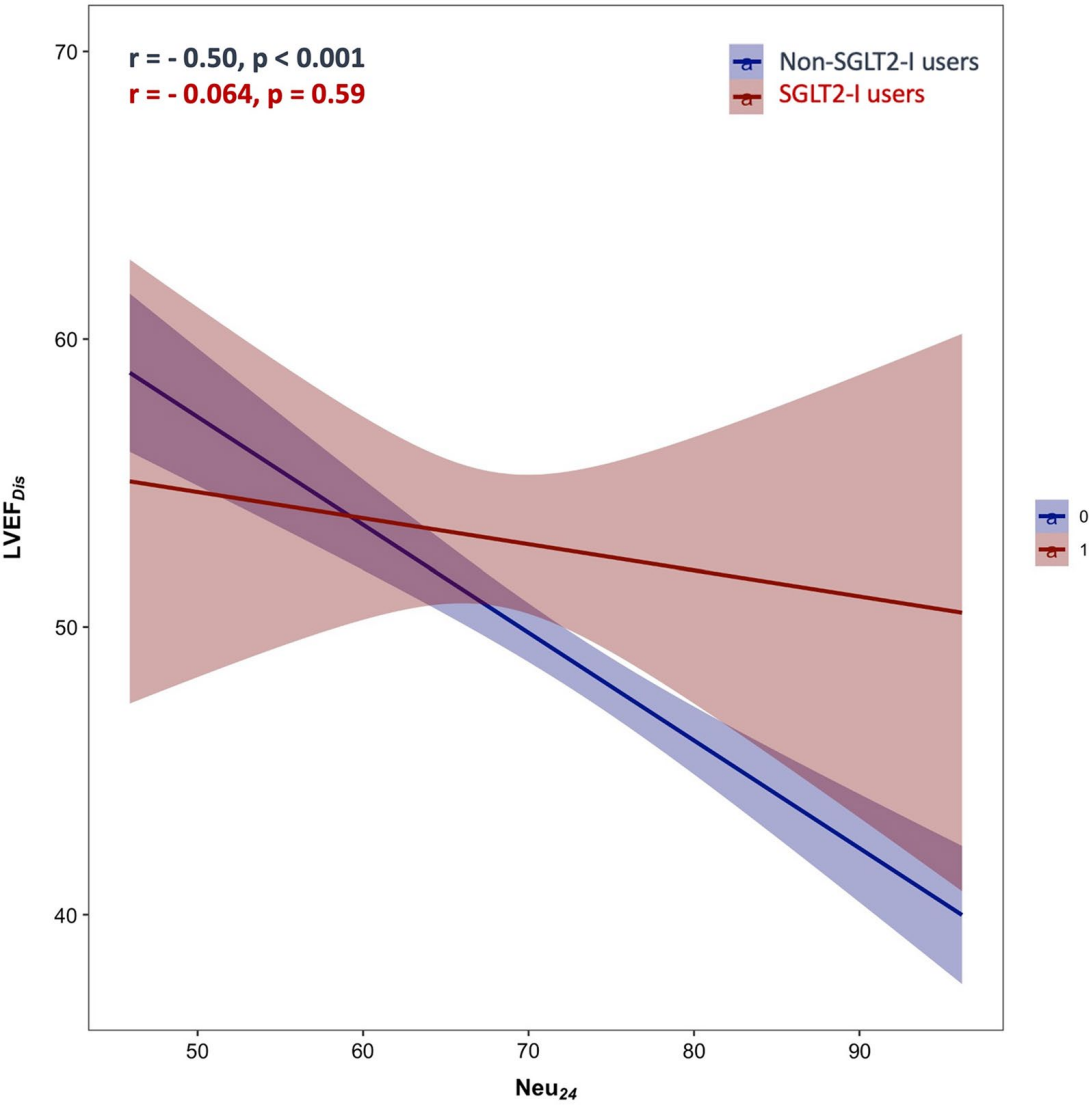
Correlation between neutrophils at 24 h and discharge LVEF in SGLT2-I versus non-SGLT2-I users (red and blue lines respectively). An inverse correlation was found between the inflammatory burden (represented by neutrophils at 24 h) and the LVEF at discharge. Conversely, in SGLT2-I users, the higher inflammatory burden was not accompanied by a reduction in LVEF at discharge

Moreover, in non-SGLT2-I patients we detected a negative linear correlation between neutrophils at 24 h and discharge LVEF (r = − 0.50 p < 0.001), a finding not confirmed in the SGLT2-I study group (Fig. 4**)**.

## Discussion

To the best of our knowledge, our study is the first aiming at investigating the protective role of SGLT2-I therapy in diabetic patients with a diagnosis of AMI. Specifically, we focused on the interplay between admission hyperglycemia, inflammation burden, and infarct size in a cohort of T2DM patients admitted with AMI, comparing SGLT2-I versus non-SGLT2-I users.

The main findings of our study are: (i) reduced infarct size was detected in patients receiving SGLT2-I compared to non-SGLT2-I patients; (ii) on admission and after 24 h, inflammatory indices were significantly higher in non-SGLT2-I users compared to the SGLT2-I group; (iii) stress hyperglycemia was significantly lower in SGLT2-I patients compared to non-SGLT2-I group, even though HbA1c did not differ between groups; (iv) the use of SGLT2-I was a significant predictor of reduced inflammatory response, independently of age, admission creatinine values and admission stress hyperglycemia; conversely, peak troponin values and NSTEMI occurrence turn out to be independent predictors of higher inflammatory status.

In the last years, SGLT2-I gained intense interest in the search for the mechanisms responsible for their beneficial effects in patients with and without DM [21]. Since SGLT2 has not been shown to be expressed in human cardiomyocytes, while it is abundantly represented in proximal tubular cells, it is intriguing how SGLT2-I might display beneficial off-target effects on the cardiovascular system. Increasing diuresis/natriuresis, improving glucose control, blood pressure-lowering, weight loss, improving vascular function, and changes in tissue sodium handling are likely to play a role [22]. In addition, some studies have hypothesized that SGLT2-I might exhibit cardiac protection beyond glucose and lipid-metabolic regulation [16, 23, 24]. Additional beneficial cardiovascular effects of SGLT2-I might include a reduction in adipose tissue-mediated inflammation and proinflammatory cytokine production, inhibition of the sympathetic nervous system, prevention of ischemia/reperfusion injury, improvement in cardiac energy metabolism with a shift towards ketone bodies as metabolic substrate, reduction of oxidative stress, and suppression of advanced glycation end-product signaling [16, 25]. Although the precise mechanisms remain unclear, immune-metabolic mechanisms have drawn increasing attention. Thus, SGLT2-I cardioprotective properties may result from both a direct effect on glucose level reduction (glucose-lowering dependent effects) and a glycemic-independent effect.

### SGLT2-I and glycemic-dependent effect

This class of antidiabetic agents has been confirmed to ameliorate glycemic parameters when used alone or in combination in T2DM patients [26]. Decreasing glucose levels by SGLT2-I may lower macrophage inflammatory response, as macrophages preferentially utilize glucose from glycolysis as an energy source [27]. In our study population, stress hyperglycemia was more frequently observed in patients treated with other OAD agents alone than in those receiving SGLT2-I. Consistent with the known interplay between stress hyperglycemia, infarct size, and inflammatory burden in AMI patients [28, 29], in our cohort we observed positive linear correlations between neutrophils values measured at 24 h and admission glucose levels and peak troponin values. According to these findings, part of the anti-inflammatory effect of this class of antidiabetic agents could be attributed to the tighter control of stress hyperglycemia, independently of HbA1c values.

### SGLT2-I and glycemic-independent effect

Whereas SGLT2-I are effective glucose-lowering agents, its cardioprotective effects are unlikely related exclusively to the improvements in glucose-lowering per se. In fact, the rapid efficacy noted (within days of treatment initiation) cannot be merely justified by a glucose-lowering mechanism [21, 22]. Likewise, in our study there were no differences in HbA1c values between the two cohorts, consistent with post hoc trial analyses showing that baseline HbA1c values are not affected by treatment with SGLT2-I [30]. A definitive proof of this concept emerged from the DAPA-HF trial, wherein the efficacy of dapagliflozin to reduce heart failure occurrence and mortality was independent of the presence of DM [13]. These data have been also confirmed in experimental models of heart failure in which the benefit of SGLT2 inhibition was observed regardless of diabetes or hyperglycemia [31, 32]. In our study, 24-hour neutrophil and peak troponin values were linearly correlated, irrespective of the admission glucose level. Moreover, SGLT2-I was identified as a significant predictor of reduced inflammatory response, regardless of admission hyperglycemia. Thus, our findings support the hypothesis of additional cardioprotective effects beyond the glucose-lowering effect *per se*, as SGLT2-I may directly target inflammatory pathways. In our study, patients previously treated with other OAD agents, compared to those receiving SGLT2-I, exhibited an amplified “inflammatory status” as expressed by increased levels of inflammatory markers (neutrophils, NLR, PLR, and CRP). Inflammation is an essential contributor to infarct size severity, and proinflammatory biomarkers correlate with the prognosis of AMI [1, 33]. Although SGLT2-I has been suggested to attenuate or ameliorate the inflammatory profile in patients with diabetes, the exact pathophysiological mechanism remains unclear [34–37]. Recent evidence suggests that empagliflozin could inhibit the nucleotide-binding domain-like receptor protein-3 (NLRP3) inflammasome and that this can occur independently of glucose-lowering per se [38, 39]. Moreover, dapagliflozin can protect from I/R damage, reduce infarct size, and improve cardiac function in non-diabetic mice, by the selective degradation of the inflammasome component NLRP3, thereby reducing maturation and secretion of inflammatory markers [40]. Part of the anti-inflammatory effects of SGLT2-I could be also related to ketone inhibition of the NLRP3 inflammasome [41, 42]. Indeed, SGLT2-I has been demonstrated to evoke a significant increase in plasma beta-hydroxybutyrate with a parallel decline in fasting plasma insulin levels due to a considerable improvement in insulin sensitivity; these effects were significantly correlated to inhibition of NLRP3 inflammasome activity [43]. Conversely, the other OAD agents (except metformin) have a prevalent B-cell secretagogues effect, leading to hyperinsulinemia/insulin resistance. The SGLT2-I anti-inflammatory properties can partially justify the reduced infarct size, as we observed in our patients. In fact, murine models of ischemia-reperfusion injury have shown that ablating the NLRP3 gene reduces the infarct size and ameliorates cardiac function [43]. Further explanations for the smaller infarct size in diabetic patients receiving SGLT2-I include the improvement in cardiac energetic metabolism triggered by this class of anti-diabetic agents. “Metabolic flexibility” is the capacity of the heart to adapt its substrate preference to short-term detrimental stimuli in order to maintain an adequate ATP production for optimal cardiac contractile function [44, 45]. In patients with T2DM, systemic and myocardial insulin-mediated glucose utilization is impaired, reducing cardiac metabolic efficiency [46]. Henceforth, in diabetic patients with stressful conditions such as AMI, further impaired glucose utilization could leave cardiomyocytes without adequate energy sources and fatty acids and ketone bodies provide an alternative energy source [42, 47]. Several studies have shown that in diabetic patients, SGLT2-I treatment exhibits protective effects by improving cardiomyocyte metabolic flexibility. Indeed, empagliflozin promotes the shift towards ketone bodies as the metabolic substrate, with a larger cardiac ATP production [48, 49]. Another beneficial effect of SGLT-2 is the improvement of sympathetic and parasympathetic nerve activity in humans [50]. Accordingly, our patients treated with SGLT-2 exhibited a lower heart rate at admission than patients treated with other OAD agents. The imbalance of the autonomic nervous system might increase myocardial ischemia, inflammation and the immune system, platelet aggregation, as well as lipoprotein and glycaemic metabolism (hyperglycaemia, hypoglycaemia, glycaemic variability).

Although the beneficial actions of SGLT2 need to be further elucidated, these agents induce many beneficial effects in multiple targets that result in a better prognosis in several cardiovascular diseases.

### Study limitations

Our results should be interpreted considering some limitations. First, laboratory parameters were incomplete in some patients, although patients with more than 20% of missing values in the collected data were excluded to avoid potential bias. Second, the sample size was powered to evaluate only a “class effect” but not the “doses effect”. Third, our study did not evaluate other inflammatory markers such as IL-6, TNF-α, IL-1, and the soluble matricellular protein cysteine-rich angiogenic inducer, which might reflect a more accurate inflammatory burden assessment. Nevertheless, a correlation between such parameters and the indices adopted in our study was previously demonstrated [6–8, 51, 52], and so we opted for measuring standardized and widely available inflammatory markers. Lastly, although we excluded patients with chronic inflammatory systemic diseases (severe valvular heart disease, severe anemia, chronic severe renal failure, autoimmune diseases, malignancies), data on chronic inflammatory conditions, before the occurrence of AMI, are lacking.

## Conclusions

Type 2 Diabetic patients hospitalized for AMI and receiving SGLT2-I exhibited a significantly reduced inflammatory response and infarct size compared to non-SGLT2-I users, independently of glucose-metabolic control. Our findings are hypothesis generating and support new pathophysiological and therapeutic insights regarding the cardioprotective effects of SGLT2-I in the setting of coronary artery disease.

## Data Availability

The datasets used and/or analysed during the current study are available from the corresponding author on reasonable request.
